# Superior Glucose Tolerance and Metabolomic Profiles, Independent of Adiposity, in HIV-Infected Women Compared With Men on Antiretroviral Therapy

**DOI:** 10.1097/MD.0000000000003634

**Published:** 2016-05-13

**Authors:** John R. Koethe, Cathy A. Jenkins, Christopher Petucci, Jeffrey Culver, Bryan E. Shepherd, Timothy R. Sterling

**Affiliations:** From the Division of Infectious Diseases (JRK, TRS); Department of Biostatistics (CAJ, BES), Vanderbilt University School of Medicine, Nashville, TN; and Sanford Burnham Prebys Metabolomics Core at the Southeast Center for Integrated Metabolomics, University of Florida (CP, JC), Gainesville, FL.

## Abstract

In epidemiologic studies, human immunodeficiency virus (HIV)-infected men on antiretroviral therapy (ART) are at higher risk of incident diabetes mellitus compared with women with similar treatment histories. We used metabolomics to determine whether a sex difference in plasma amino acids, acylcarnitines, and organic acids predictive of diabetes and impaired energy metabolism is present in HIV-infected persons on long-term ART.

We enrolled 70 HIV-infected adults (43% women) on efavirenz, tenofovir, and emtricitabine (Atripla) with HIV-1 RNA <50 copies/mL for over 2 years. Half of the HIV-infected subjects were obese, and these were matched with 30 obese HIV-negative controls. All subjects had no history of diabetes, statin use, or heavy alcohol use. Fasting insulin sensitivity was measured using homeostatic model assessment 2 (HOMA2), and adipose tissue was measured using dual-energy x-ray absorptiometry (DEXA). Liquid chromatography/mass spectrometry was used to quantitate fasting plasma branched chain and aromatic amino acids predictive of incident diabetes, and C3 and C5 acylcarnitinines and organic acids indicative of impaired energy metabolism.

HIV-infected women had more baseline risk factors for insulin resistance: women were older (46 vs 44 years) and had a longer ART duration (8.4 vs 5.1 years, *P* < 0.05 for both) compared with men but had similar CD4+ count (median 701 cells/μL), smoking and hepatic C prevalence, and body mass index (BMI) (median 30.3 kg/m^2^). However, women had higher insulin sensitivity compared with men (*P* < 0.01), and lower plasma levels of isoleucine, leucine, valine, phenylalanine, and tyrosine (*P* < 0.01 for all), and lower C3 and C5 acylcarnitines (*P* < 0.01 for all), in multivariable regression models after adjusting for DEXA fat mass index, age, race, CD4+ count, smoking, and ART duration. In the obese HIV-infected subjects and HIV-negative controls, the relationship of sex and plasma metabolite levels did not significantly differ according to HIV-status.

HIV-infected women on non-nucleoside reverse transcriptase inhibitor-based ART had superior glucose tolerance and lower plasma metabolites associated with the development of diabetes compared with men with similar metabolic disease risk profiles. The relationship between sex and plasma metabolite levels did not significantly differ according to HIV-status among obese subjects, suggesting the observed sex-differences may not be specific to HIV infection.

## Abstract 2

Supplemental Digital Content is available in the text

## INTRODUCTION

Cardiometabolic disease is increasingly prevalent among human immunodeficiency virus (HIV)-infected individuals on long-term antiretroviral therapy (ART), and several epidemiologic analyses have shown that HIV-infected men are at higher risk of developing diabetes compared with women.^1–4^ In a study of HIV-infected veterans, men had a 2-fold higher risk of prevalent diabetes compared with women,^3^ while in the Data Collection on Adverse Events of Anti-HIV Drugs Study (D:A:D) men had a 1.6-fold higher risk of incident diabetes.^5^ Studies of the general population have also found diabetes to be more prevalent among men, though the risk associated with male sex is generally lower compared with the risk reported in HIV cohort studies.^6–8^ At present, it is unclear whether the higher risk of diabetes in HIV-infected men represents a true sex-difference, or reflects confounding related to ART treatment (e.g., duration of treatment, the composition of the ART regimen) or other factors (e.g., body mass index [BMI], body fat content, or alcohol use). Interpreting the epidemiologic literature is hampered by a lack of clinical data on energy metabolism in HIV-infected men versus women with similar ART exposure and non-HIV risk profiles.

Metabolomics—the identification of metabolite profiles reflective of cellular processes—presents an opportunity to assess energy metabolism abnormalities in greater detail than glucose tolerance testing alone. Plasma levels of amino acids and other small molecules reflective of impaired energy metabolism, such as acylcarnitines (intermediary metabolites produced from amino acids, fatty acids, and other energy sources) and organic acids, can be reliably measured using mass spectrometry to provide a detailed metabolic profile.

In Framingham and other cohorts, a plasma metabolite profile characterized by elevated branched chain amino acids (BCAA; leucine, isoleucine, and valine), the aromatic amino acids phenylalanine and tyrosine, and C3 and C5 acylcarnitines (BCAA degradation products) could identify individuals who subsequently developed diabetes mellitus, more than 10 years prior to clinical disease onset in some cases.^9–11^ A postulated mechanism underlying this finding is the toxic effect of high plasma BCAA and related metabolites on mitochondrial fitness in muscle and other tissues, resulting in reduced glucose uptake.^12–14^ Similar cohort studies have also implicated several organic acids as indictors of impaired glucose tolerance and diabetes risk, including 2-hydroxybutyrate, 3-hydroxybutyrate, and lactate.^15,16^

Recent metabolite profiling studies in HIV-negative individuals suggest that women are better able to maintain insulin sensitivity compared with men, but at present there are no such data available in HIV-infected persons. Given the striking sex difference in incident diabetes risk observed in HIV cohort studies, we used metabolomics to characterize phenotypic differences in energy metabolism between healthy, non-diabetic HIV-infected men and women through quantification of plasma amino acids, acylcarnitines, and organic acids previously shown to reflect impaired energy metabolism. Furthermore, we compared HIV-infected subjects and matched HIV-negative controls to determine if sex differences in metabolite levels were specific to HIV infection.

## METHODS

### Participant Recruitment

We enrolled 70 HIV-infected patients on ART from the Vanderbilt Comprehensive Care Clinic between April 2013 and September 2014.^17^ Participants were distributed equally between four BMI categories (<25.0, 25.0–29.9, 30.0–34.9, and ≥35.0 kg/m^2^), and within each category an approximately equal number of men and women, and whites and non-whites, were enrolled. All subjects were on efavirenz, tenofovir, and emtricitabine (i.e., the combination pill *Atripla*) for at least the 6 months prior to enrollment and had been on ART with consistent HIV-1 RNA measurements <50 copies/mL for at least the previous 2 years. Additional inclusion criteria were CD4+ count >350 cells/μL at the time of enrollment, no use of any anti-diabetic medication or statin (i.e., HMG CoA reductase inhibitor) in the prior 6 months, no self-reported heavy alcohol (defined as >11 drinks/week) or cocaine/amphetamine use, no active infectious conditions aside from HIV, and no previously diagnosed diabetes, cardiovascular disease, or rheumatologic disease.

Thirty HIV-negative volunteers with obesity were recruited from the community to serve as controls. Controls were distributed equally between the BMI categories of 30.0–34.5 and >35 kg/m^2^ and group-matched by sex and race with the HIV-infected subjects. The HIV-negative controls had not received any anti-diabetic medications or statins in the prior 6 months, did not report alcohol or illicit drug abuse, and had no active infectious conditions or previously diagnosed diabetes, cardiovascular disease, or rheumatologic disease.

### Clinical Assessment

Data on ART and other medication history, CD4+ count and HIV-1 RNA values, and hepatitis C antibody status were obtained from the medical record; smoking status was by self-report. All subjects underwent an assessment in the Vanderbilt Clinical Research Center after fasting overnight for at least 8 hours (all visits began between 8 and 11 am). Prior to any interventions, fasting blood (10 mL) was collected in an EDTA vacutainer, immediately centrifuged for 10 minutes at 4 °C, and the plasma removed and frozen at −80 °C for metabolomic profiling. Fasting plasma glucose and insulin were measured and beta cell function and insulin sensitivity were calculated using the Homeostasis Model Assessment 2 (HOMA2) equation (https://www.dtu.ox.ac.uk/homacalculator).^18^ Weight was determined using an electronic scale, height using a stadiometer fixed to the wall, and waist circumference using a flexible tape measure parallel to the floor at a point 1 inch above the navel.

A full body dual-energy x-ray absorptiometry (DEXA) scan was performed using a GE Healthcare Lunar iDXA and analyzed using enCORE software version 13.6 (GE Healthcare Lunar, Madison, Wisconsin). Total fat mass was used to calculate fat mass index (FMI; defined as DEXA total fat in kilograms divided by height in meters squared), which compensates for the non-linear relationship between fat-free mass and height, and provides a more accurate estimation of relative adiposity compared with BMI or percentage body fat.^19,20^ Android fat mass (located at the abdomen) was calculated by the enCORE software as the area from the pelvis cut (the upper edge of the iliac crest) extending superiorly one-fifth the distance from the pelvis cut to the neck cut.^21^ The upper border of the gynoid fat mass (located at the hips) began at a point 1.5-times the height of the android region below the pelvis cut and extended inferiorly an additional 2-times the height of the android region. Lastly, visceral fat mass was calculated by subtracting subcutaneous android fat mass (derived using data on total abdominal thickness, the width of the subcutaneous fat layer along the lateral extent of the abdomen, and empirically derived geometric constants) from total android fat mass.^22^ This method correlated well with (computed tomography measurements of visceral fat in a validation study.^22^

### Extraction and Derivatization of Amino Acids and Acylcarnitines

Using frozen, fasting samples, a 100 μL aliquot of human plasma was spiked with a 10 μL mixture of amino acid or acylcarnitine internal standard mixture and treated with bleach (to inactivate HIV particles) to a final concentration of 10% bleach. Samples were treated with 800 μL of ice-cold methanol and centrifuged to pellet precipitated protein. Amino acids were derivatized by drying down 100 μL of methanolic extract and reconstituting the sample with 80 μL of borate buffer (Waters Corp., Milford, MA) and 20 μL of MassTrak AAA Reagent Powder dissolved in MassTrak AAA Reagent Diluent (Waters Corp., Milford, MA) in a 96-well plate. Acylcarnitines were derivatized by reconstituting the dried methanolic extract in 100 μL of 0.2 M O-benzylhydroxylamine (Sigma–Aldrich, St. Louis, MO) and 10 μL of 2 M 1-ethyl-3-(3-dimethylaminopropyl) carbodiimide (Sigma–Aldrich, St. Louis, MO).

### Extraction and Derivatization of Organic Acids

A 50 μL aliquot of human plasma was spiked with 10 μL of a mixture of organic acid internal standards and bleach (final concentration of 10%) followed by the addition of 50 μL of 0.4 M O-benzylhydroxylamine and 10 μL of 2 M 1-ethyl-3-(3-dimethylaminopropyl) carbodiimide. Samples were derivatized at room temperature for 20 minutes, and organic acids were extracted from the plasma by liquid-liquid extraction using ethyl acetate. Samples were centrifuged to create a bilayer. A 100-μL aliquot of the ethyl acetate layer was dried and reconstituted in 1 mL of 50/50 methanol/water prior to analysis.

### Liquid Chromatography/Mass Spectrometry Quantification

Derivatized amino acids (0.5-μL injection) were separated on a 2.1 × 100 mm, 1.7-μm Waters AccQ-Tag Ultra C18 column maintained at 55 °C using a 9.55-minute linear gradient with aqueous and organic mobile phases proprietary to Waters Corp. at a flow rate of 0.7 mL/min. Derivatized acylcarnitines (2-μL injection) were separated on a 2.1 × 100 mm, 1.7 μm Waters Acquity UPLC BEH C18 column maintained at 50 °C using a 12.5-minute linear gradient with 0.1% formic acid in water and 0.1% formic acid in acetonitrile at a flow rate of 0.4 mL/min. Quantification of derivatized amino acids and acylcarnitines was achieved using multiple reaction monitoring on an Agilent 1290 Infinity HPLC/6490 triple quadrupole mass spectrometer (Agilent, Wilmington, DE).

Derivatized organic acids were separated on a 2.1 × 100 mm, 1.7 μm Waters Acquity UPLC BEH C18 column (*T* = 45 °C) using a 7.5-minute linear gradient with 0.1% formic acid in water and 0.1% formic acid in acetonitrile at a flow rate of 0.3 mL/min. Quantification of derivatized organic acids was achieved using multiple reaction monitoring on an Dionex UltiMate 3000 HPLC/Thermo Scientific Quantiva triple quadrupole mass spectrometer (Thermo Scientific, San Jose, CA).

Standard calibration curves for the derivatized metabolites were prepared using commercially available amino acids (Sigma–Aldrich, St. Louis, MO) and internal standards (Sigma–Aldrich, St. Louis, MO; CDN Isotopes, Quebec, Canada); acylcarnitines (Sigma–Aldrich, St. Louis, MO; Toronto Research Chemicals, Toronto, Canada; Larodan, Solna, Sweden; R&D Systems, Minneapolis, MN) and internal standards (Sigma–Aldrich, St. Louis, MO; Toronto Research Chemicals, Toronto, Canada; Larodan, Solna, Sweden); and organic acids (Sigma–Aldrich, St. Louis, MO) and internal standards (Sigma–Aldrich, St Louis, MO; Cambridge Isotopes, Cambridge, MA; CDN Isotopes, Quebec, Canada).

### Statistical Analyses

Demographics, clinical characteristics, and plasma levels of amino acids, acylcarnitines, and organic acids for HIV-infected men and women were expressed as medians and interquartile range or percentages and compared using Wilcoxon rank sum or Chi square tests as appropriate. Multivariable regression models to assess the effect of sex on glucose tolerance and each plasma metabolite were adjusted for age, race (white versus non-white), ART duration, current smoking status, CD4+ count (square root transformed), and FMI. An interaction term between sex and FMI was included in the models to assess whether FMI modified the relationship between sex and the outcomes; the term was removed from the final model for each outcome if the *P*-value for interaction was not significant (*P* > 0.05). Model diagnostics were assessed using residual/predictor plots and outcome variables were log-transformed as needed (HOMA2 insulin sensitivity, HOMA2 beta cell function, and 3-hydroxybutyric acid required transformation). C5 valeryl was excluded from the multivariable analyses as 29 subjects (41%) had a level below the 0.0025 μM limit of detection.

Sensitivity analyses were performed which replaced FMI with BMI. We fit additional, individual regression models, which further adjusted for calculated visceral fat, waist circumference, and gynoid fat mass, in addition to separate models, which adjusted for pre-treatment CD4+ count and a history of an AIDS-defining event before ART initiation.

We also compared obese HIV-infected and HIV-negative participants according to demographics, clinical characteristics, and plasma metabolite levels using Wilcoxon rank sum or Chi square tests as appropriate. To assess whether the relationship between sex and the primary metabolites of interest differed according to HIV status we fit a multivariable regression model with the following covariates: age, sex, HIV-status, race, current smoking status, FMI and an interaction term for sex and HIV-status; the term was removed from the final model for each outcome if the *P*-value for interaction was not significant (*P* > 0.05).

The primary metabolites of interest (leucine, isoleucine, valine, phenylalanine, and tyrosine, C3 and C5 acylcarnitines, and 2-hydroxybutyrate, 3-hydroxybutyrate, and lactate) were pre-selected based on prior studies in the literature and therefore no adjustments were made for multiple comparisons.^23^ The cohort sample size was selected to provide adequate power for detecting associations between fat mass and several serum biomarkers of inflammation among the HIV-infected subjects, which pre-enrollment calculations also indicated would be sufficient to detect a sex difference in the primary metabolite outcomes in this analysis. Analyses were conducted using SPSS 22.0.0 (IBM, Armonk, New York) and R Statistical Software (http://www.R-project.org).

The study was reviewed and approved by the Vanderbilt University Institutional Review Board. All participants provided written informed consent.

## RESULTS

The clinical and demographic characteristics of the HIV-infected subjects are shown in Table 1. HIV-infected women were slightly older than men, more likely to be non-white, and had been on ART for a longer period (*P* < 0.05 for all), but there was no difference in median CD4+ cell count at enrollment, or prevalence of smoking or hepatitis C coinfection. While the median BMI, waist circumference, android fat mass, and calculated visceral fat did not differ by sex, women had a higher FMI and gynoid fat mass.

**TABLE 1:**
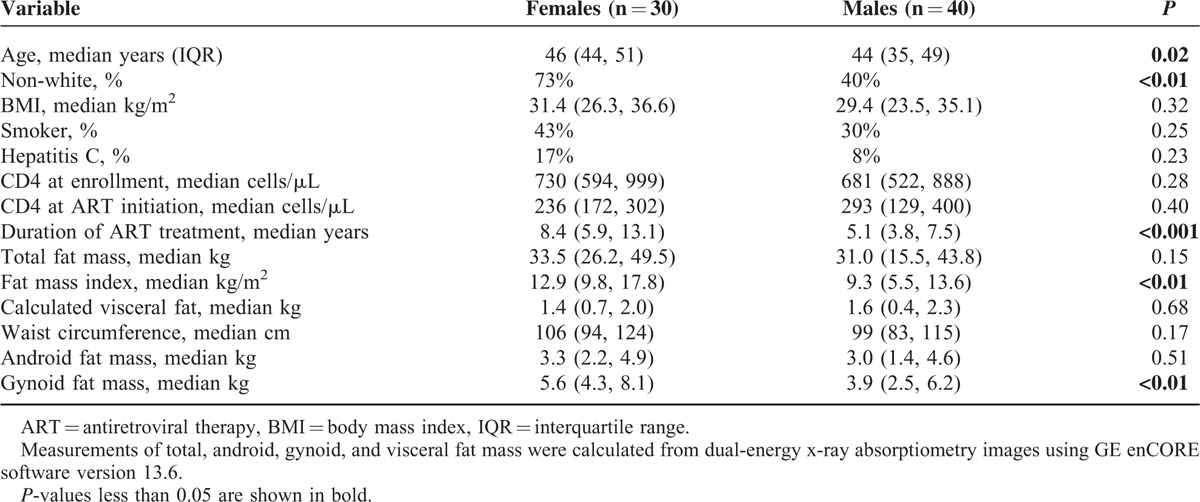
Clinical Characteristics and Body Composition of Female and Male HIV-Infected Subjects

Despite being older, on ART for a longer period, and having a higher FMI, women had similar median HOMA2 insulin release and sensitivity values (Table 2). Furthermore, women had lower median plasma isoleucine, leucine, valine, and phenylalanine levels than men (*P* ≤ 0.01 for all), and lower C3 and C5 acylcarnitine values compared with men (*P* < 0.001 for all), but no significant difference in tyrosine or the organic acids (the *P*-value for 3-hydroxybutyrate was 0.05). Median values for all amino acids, acylcarnitines, and organic acids measured in our study are shown in Supplementary Table 1; the metabolite sex differences were most consistent and pronounced in the BCAA, aromatic amino acids, and C3/C5 acylcarnitines previously found to be associated with diabetes risk.

**TABLE 2:**
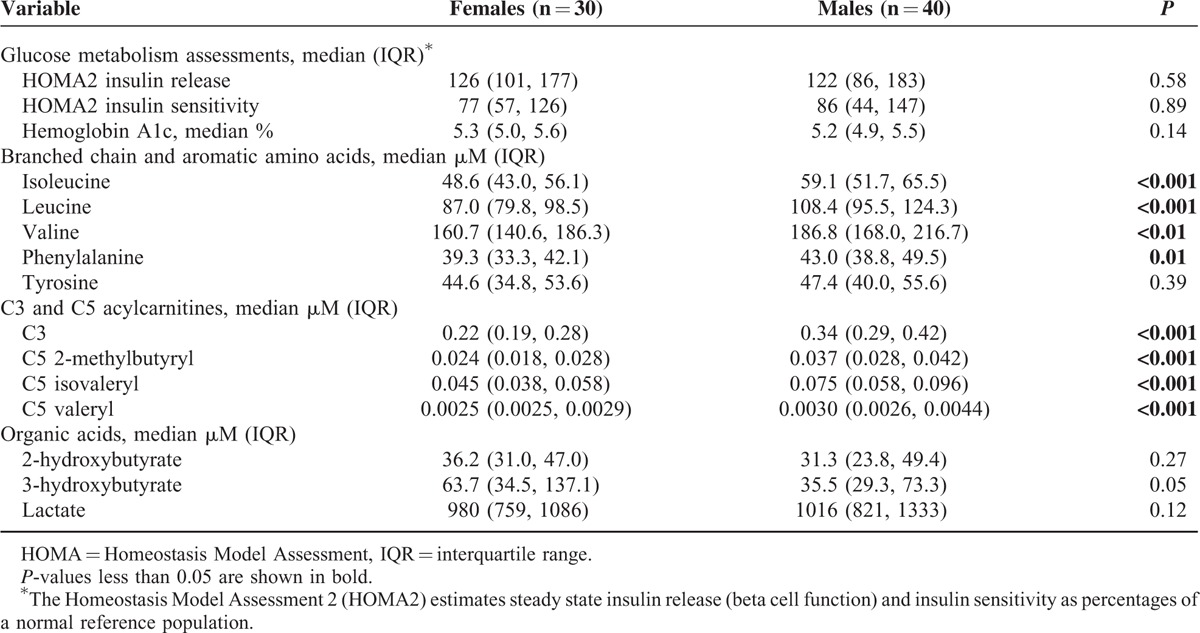
Unadjusted Comparison of Glucose Tolerance and Plasma Metabolites Associated with Diabetes Risk Between Female and Male HIV-Infected Subjects

In the multivariable models, female sex was associated with lower HOMA2 insulin release and higher insulin sensitivity, lower isoleucine, leucine, valine, phenylalanine, and tyrosine levels, and lower C3 and C5 acylcarnitines (*P* < 0.01 for all, Table 3). However, we did not observe a difference in 2-hydroxybutyrate, 3-hydroxybutyrate, or lactate between sexes. The interaction term for FMI and sex was significant only for the regression models with HOMA2 insulin sensitivity and insulin release, and therefore the interaction term was not included in models for the remaining outcomes. HIV-infected women as a group had greater insulin sensitivity compared with men (β = 0.184, *P* < 0.001), and the reduction in insulin sensitivity for each unit (kg/m^2^) increase in FMI was smaller for women compared with men (−0.017 vs −0.055, *P*-value for interaction <0.001). Similarly, women had lower insulin release (β = −0.09, *P* < 0.001) and the rise in insulin levels per FMI unit was lower among women compared with men (0.009 versus 0.038, *P*-value for interaction <0.001). The results of the multivariable models for the primary outcomes were similar after replacing FMI with BMI, and after adjusting for calculated visceral fat, gynoid fat mass, waist circumference, pre-treatment CD4+ count, and a history of an AIDS-defining event prior to ART initiation in separate sensitivity analyses.

**TABLE 3:**
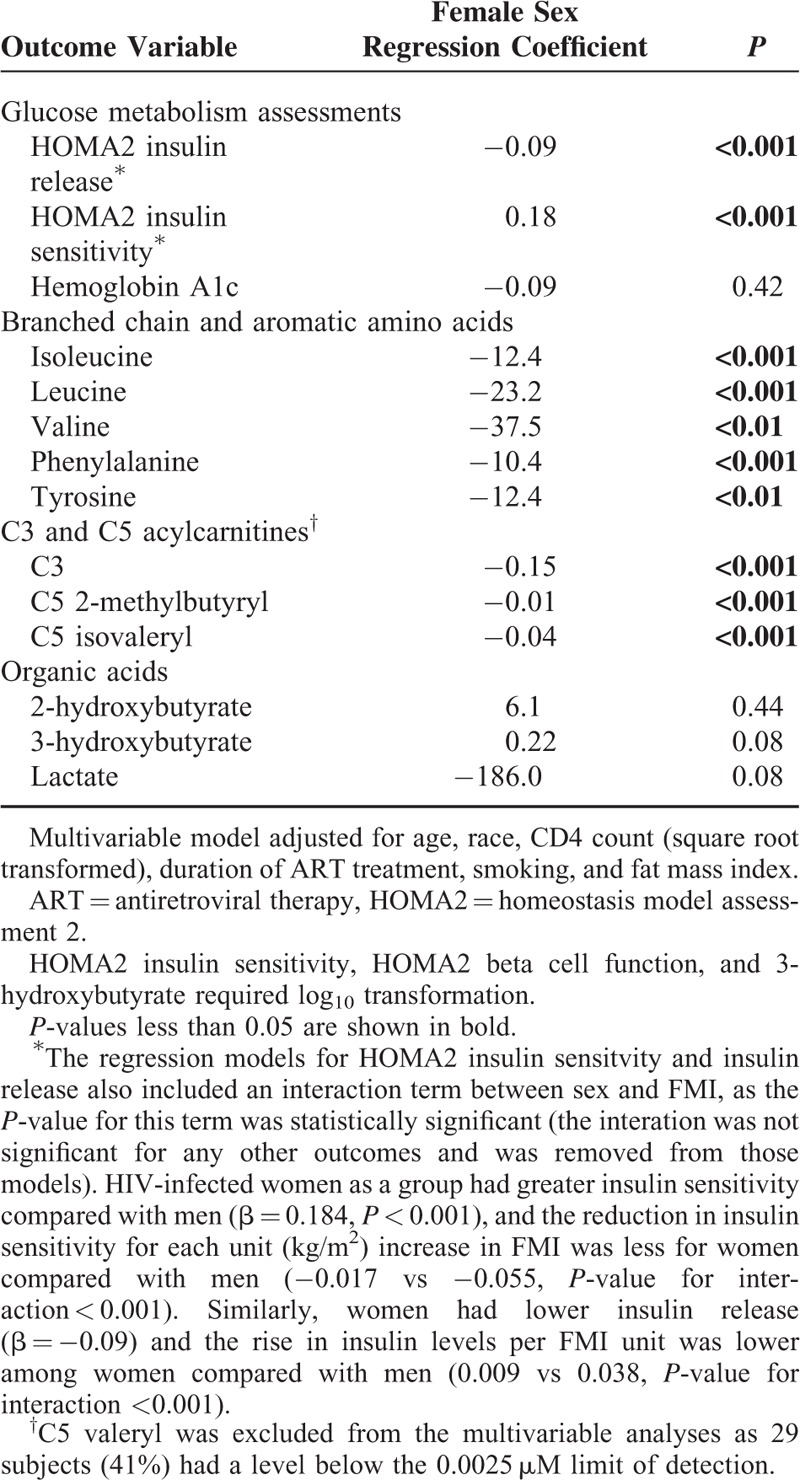
Multivariable Linear Regression Model for the Relationship of Sex With Glucose Metabolism and Plasma Metabolites Indicative of Diabetes Risk in HIV-infected Subjects on Long-Term ART (n = 70)

Lastly, we assessed whether sex-differences in insulin sensitivity and metabolite levels differed according to HIV status in the obese HIV-infected subjects (n = 35) and the obese HIV-negative controls (n = 30). The obese HIV-infected subjects had a higher median age compared with the obese controls, and a higher smoking and hepatitis C prevalence, but did not significantly differ according to sex, race, BMI, FMI, or HOMA2 insulin sensitivity (Table 4). Additionally, there were no significant differences in median levels of isoleucine, leucine, valine, phenylalanine, tyrosine, C3 and C5 acylcarnitines, or organic acids between obese HIV-infected subjects and obese HIV-negative controls, though several acylcarnitines derived from fatty acid oxidation (e.g., C12 and C14) were higher in the HIV-infected subjects (Supplementary Table 2).

**TABLE 4:**
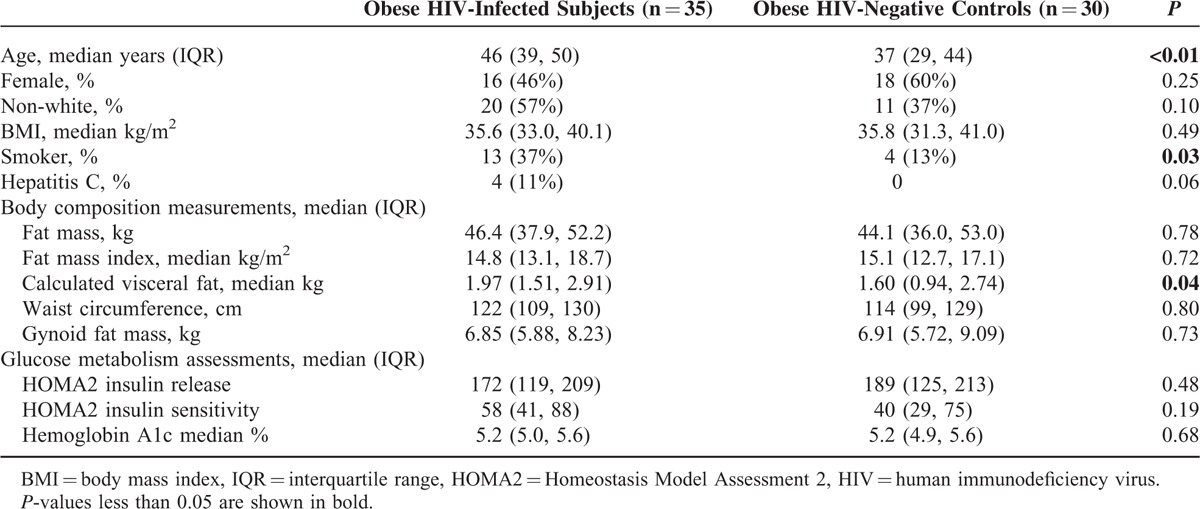
Clinical Characteristics, Body Composition, and Glucose Tolerance in Obese HIV-infected Subjects Versus Obese HIV-negative Controls

In the multivariable model pooling the obese HIV-infected subjects and HIV-negative controls, the pattern of statistical associations between female sex and lower plasma metabolite levels was similar to that observed in the HIV-infected participants alone (Table 5). Furthermore, we did not detect a significant interaction between HIV-status and sex for any primary outcome variables except HOMA2 insulin release and C5 isovaleryl, indicating the relationship between sex and the outcome was similar in both the HIV-infected subjects and HIV-negative controls.

**TABLE 5:**
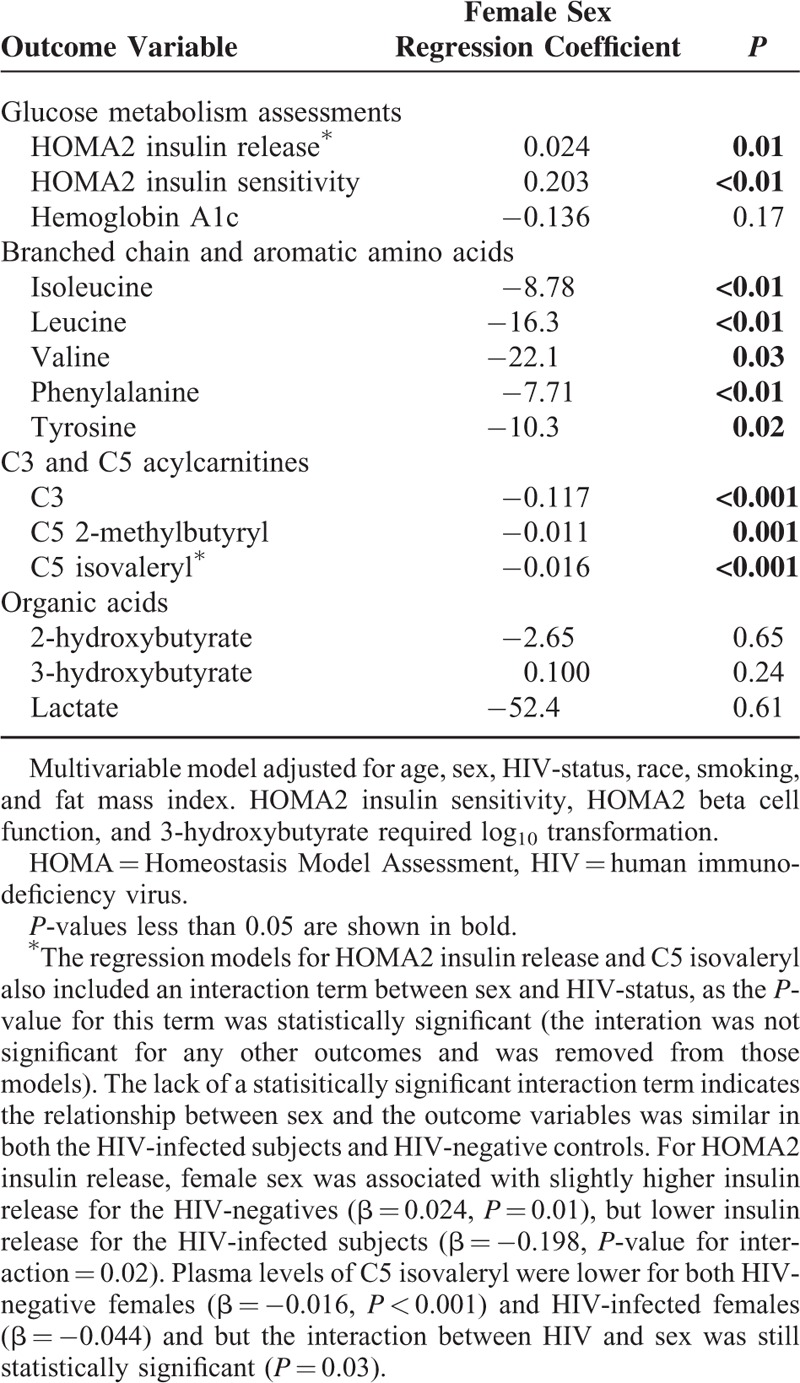
Multivariable Linear Regression Model for the Relationship of Sex With Glucose Metabolism and Plasma Metabolites in Obese HIV-infected Subjects and Obese HIV-negative Controls (n = 65 Total Subjects)

## DISCUSSION

HIV-infected women on long-term, non-nucleoside reverse transcriptase-based ART without previously diagnosed metabolic disease had significantly better glucose tolerance and a plasma metabolite signature associated with a lower likelihood of progression to diabetes compared with men, which was independent of age, race, adiposity, and other known risk factors. This finding provides clinical evidence of a sex difference in diabetes risk among HIV patients on ART, which is in accordance several large epidemiologic analyses showing markedly higher rates of incident diabetes in HIV-infected men compared with women.^3,5,24^ However, with the exception of HOMA2 insulin release and plasma levels of C3 isovaleryl, we observed no significant difference in the relationship of sex and metabolite levels according to HIV-status among the obese HIV-subjects and obese HIV-negative controls, suggesting that sex-related biological differences rather than HIV-specific factors may underlie our findings.

We believe this is the first metabolite profiling study of HIV-infected men and women on long-term ART, but a few similar analyses have been performed in HIV-negative cohorts. In a study of 73 overweight and obese subjects (48% women), men had significantly higher plasma leucine, isoleucine, valine, phenylalanine, proline, glutamate/glutamine, and methionine levels, and higher C3 and C5 acylcarnitines, compared with women, but no difference in 3-hydroxybutyrate or lactate.^13^ Similarly, in a European cohort of healthy volunteers, men (n = 35) had significantly higher plasma leucine, isoleucine, valine, tyrosine, and other amino acid levels, but no difference in C3 or C5 acylcarnitine levels, compared with women not on oral contraceptives (n = 41).^25^ However, women on oral contraceptives (n = 26; containing ethinylestradiol and progestins) had higher isoleucine levels compared with women not on oral contraceptives, which suggests sex hormones may impact some metabolite pathways (a hypothesis supported by animal studies).^26,27^ In our cohort, five women reported oral contraceptive use and a similar number reported receiving depo-provera, but for most recipients these were provided by a separate clinic and we could not confirm refill or injection dates. Due to this uncertainty we did not assess the effect of hormonal contraception on our outcomes. Lastly, a recent study of obese teenagers (41 men and 41 women, mean BMI 35 kg/m^2^ for both sexes, none with known HIV) found men had higher fasting levels of BCAA and C3 and C5 acylcarnitines, but no difference in phenylalanine or tyrosine.^28^ Of note, the authors report higher levels of glutamic acid in men, which we also observed in our cohort.

The sex differences we observed in branched chain and aromatic amino acids, and C3 and C5 acylcarnitines, were not accompanied by similar differences in organic acids. In particular, 2-hydroxybutyrate, a strong predictor of diabetes risk in the Relationship of Insulin Sensitivity to Cardiovascular Risk study, did not differ by sex.^15^ We also did not observe a difference in plasma lactate, a marker of insufficient oxidative capacity to meet energy requirements, which was associated with prevalent diabetes in the Atherosclerosis Risk in Communities study.^16^ We hypothesize that in our cohort, metabolic abnormities had not yet reached a point where fasting energy requirements grossly exceeded oxidative capacity.

The finding that median HOMA2 insulin sensitivity and median levels of most plasma metabolites associated with diabetes risk were not significantly different in the obese HIV-infected subjects versus HIV-negative controls was unexpected in light of epidemiologic studies showing significantly higher rates of incident diabetes in HIV-infected patients compared with non-infected persons.^2,3^ We attribute this to 3 factors. First, prior studies included many HIV patients with substantial cumulative exposure to stavudine and zidovudine, which are independently associated with greater glucose intolerance as compared with the combination of efavirenz, tenofovir, and emtricitabine in our cohort (few subjects had any exposure to other ART agents).^2,5^ Second, we postulate that effects of excess adiposity on glucose metabolism among obese individuals may mask any additive impact from HIV infection or ART regimen, and a difference may have been apparent if we compared non-obese HIV-infected adults to non-obese controls. Third, the size of our control arm may have limited statistical power. In contrast, several acylcarnitines derived from fatty acid oxidation were significantly higher in the HIV-infected subjects, likely a reflection of the hypertricylceridemia and other lipid abnormalities common in HIV infection.^29–31^

Strengths of our study included a relatively uniform cohort without known cardiometabolic disease, and all HIV-infected subjects were on the same ART regimen with viral suppression for over 2 years. Fewer obese control subjects (n = 30) were enrolled than obese HIV-infected subjects (n = 35), which may have limited our statistical power. However, the controls were matched to HIV-infected subjects by sex, race, and BMI during enrollment, and met the same criteria for excluded medications and alcohol use to reduce confounding. All HIV-infected subjects in our study were on efavirenz, tenofovir, and emtricitabine, a regimen which has been associated with higher blood glucose levels,^32,33^ and we could not assess how this particular ART regimen might affect overall metabolite levels or metabolite differences between men and women. In particular, additional studies are needed to determine whether similar findings are present in patients on protease inhibitor and integrase inhibitor-based regimens. Lastly, the design of our study did not permit a comparison of non-obese HIV-infected and HIV-negative individuals.

In conclusion, we found that female HIV patients on non-nucleoside reverse transcriptase-based combination ART with long-term virologic suppression had superior glucose tolerance and lower plasma metabolomic biomarkers associated with diabetes risk compared with male patients on the same regimen. These findings provide clinical evidence of sex differences in metabolic fitness in the context of HIV treatment which may explain the male predominance in incident diabetes reported in epidemiologic analyses. The relationship between sex and metabolite levels did not differ by HIV-status in obese patients, suggesting sex-related biological differences rather than HIV-specific factors may underlie our findings. Further studies are needed to explore the sex-related determinants of metabolic fitness and the impact on the development of HIV-associated diabetes.

## Supplementary Material

Supplemental Digital Content
