# Supplementary material for: Superior Glucose Tolerance and Metabolomic Profiles, Independent of Adiposity, in HIV-Infected Women Compared With Men on Antiretroviral Therapy
Source: Medicine (Baltimore). 2016 May 13;95(19):e3634. doi: 10.1097/MD.0000000000003634 (PMC4902518; doi:10.1097/MD.0000000000003634)
Supplement: Supplemental Digital Content [file medi-95-e3634-s001.doc]

| **Supplementary Table 1. Unadjusted comparison of all measured plasma amino acids, acylcarnitines, and organic acids between female and male HIV-infected subjects (n=70)** | | | |
| --- | --- | --- | --- |
| **Plasma metabolite** | **Females (n=30)** | **Males (n=40)** | **p-value** |
| **Amino acids, median µM (IQR)** | | |  |
| Alanine | 263 (233, 301) | 274 (236, 317) | 0.44 |
| Arginine | 29.9 (25.5, 33.0) | 28.6 (22.4, 32.7) | 0.27 |
| Asparagine | 32.9 (29.9, 35.1) | 33.6 (30.6, 38.6) | 0.33 |
| Aspartic acid | 3.81 (2.67, 5.60) | 4.12 (2.79, 5.38) | 0.65 |
| Citrulline | 30.3 (25.9, 37.6) | 30.2 (25.5, 34.2) | 0.76 |
| Glutamic acid | 29.4 (27.3, 43.2) | 41.8 (34.6, 59.1) | **0.03** |
| Glutamine | 359 (295, 441) | 347 (304, 413) | 0.46 |
| Glycine | 227 (187, 261) | 210 (181, 262) | 0.66 |
| Histidine | 66.8 (61.1, 71.1) | 71.3 (62.9, 76.2) | 0.10 |
| Isoleucine | 48.6 (43.0, 56.1) | 59.1 (51.7, 65.5) | **<0.001** |
| Leucine | 87.0 (79.8, 98.5) | 108.4 (95.5, 122.3) | **<0.001** |
| Lysine | 195 (152, 218) | 210 (180, 238) | 0.20 |
| Ornithine | 39.2 (33.6, 47.2) | 44.4 (36.4, 51.8) | 0.22 |
| Phenylalanine | 39.3 (33.3, 42.1) | 43.0 (38.8, 49.5) | **0.01** |
| Proline | 118 (96, 143) | 141 (120, 171) | **<0.01** |
| Serine | 73.4 (63.6, 85.9) | 70.3 (59.4, 80.9) | 0.40 |
| Threonine | 99.9 (82.0, 128.2) | 97.4 (84.0, 118.8) | 0.59 |
| Tryptophan | 27.3 (23.6, 32.8) | 33.3 (27.8, 38.3) | **0.02** |
| Tyrosine | 44.6 (34.8, 53.6) | 47.4 (40.0, 55.6) | 0.39 |
| Valine | 160.7 (140.6, 186.3) | 186.8 (168.0, 216.7) | **<0.01** |
| **Acylcarnitines, median µM (IQR)** | | |  |
| C2 | 7.55 (5.62, 10.85) | 5.17 (4.41, 7.41) | **<0.01** |
| C3 | 0.22 (0.19, 0.28) | 0.34 (0.29, 0.42) | **<0.001** |
| C4 Butyryl | 0.070 (0.052, 0.083) | 0.075 (0.055, 0.104) | 0.32 |
| C4 Isobutyryl | 0.050 (0.036, 0.068) | 0.071 (0.038, 0.096) | 0.22 |
| C4-DC Succinyl | 0.0077 (0.0050, 0.0089) | 0.0081 (0.0066, 0.0114) | 0.20 |
| C4-OH Butyryl | 0.0085 (0.0050, 0.0172) | 0.0050 (0.0050, 0.0124) | **0.04** |
| C5 2-methylbutyryl | 0.024 (0.018, 0.028) | 0.037 (0.028, 0.042) | **<0.001** |
| C5 isovaleryl | 0.045 (0.038, 0.058) | 0.075 (0.058, 0.096) | **<0.001** |
| C5 valeryl | 0.0025 (0.0025, 0.0029) | 0.0030 (0.0026, 0.0044) | **< 0.001** |
| C5:1 | 0.0038 (0.0030, 0.0054) | 0.0057 (0.0041, 0.0073) | **<0.01** |
| C5-OH | 0.0067 (0.0052, 0.0078) | 0.0099 (0.0074, 0.0122) | **<0.001** |
| C6 | 0.037 (0.028, 0.048) | 0.029 (0.023, 0.037) | **0.03** |
| C8 | 0.101 (0.073, 0.152) | 0.080 (0.060, 0.122) | 0.05 |
| C8-OH | 0.017 (0.012, 0.021) | 0.016 (0.012, 0.023) | 0.91 |
| C10 | 0.105 (0.072, 0.160) | 0.081 (0.051, 0.115) | **0.04** |
| C10-OH | 0.049 (0.039, 0.066) | 0.052 (0.036, 0.070) | 0.80 |
| C12 | 0.046 (0.031, 0.073) | 0.043 (0.033, 0.052) | 0.38 |
| C14:1 | 0.065 (0.044, 0.106) | 0.052 (0.038, 0.079) | 0.10 |
| C14:2 | 0.033 (0.021, 0.043) | 0.025 (0.017, 0.038) | 0.17 |
| C14 | 0.021 (0.015, 0.027) | 0.018 (0.015, 0.023) | 0.70 |
| C16:2 | 0.0089 (0.0059, 0.0135) | 0.0063 (0.0046, 0.0094) | 0.05 |
| C16 | 0.068 (0.061, 0.090) | 0.076 (0.066, 0.089) | 0.61 |
| C18:1 | 0.123 (0.094, 0.141) | 0.112 (0.092, 0.133) | 0.35 |
| C18:2 | 0.091 (0.073, 0.108) | 0.097 (0.074, 0.121) | 0.39 |
| C18 | 0.031 (0.026, 0.035) | 0.035 (0.029, 0.042) | **0.04** |
| **Organic acids, median µM (IQR)** | | |  |
| 2-hydroxybutyrate | 36.2 (31.0, 47.0) | 31.3 (23.8, 49.4) | 0.27 |
| 3-hydroxybutyrate | 63.7 (34.5, 137.1) | 35.5 (29.3, 73.3) | 0.05 |
| α-ketoglutarate | 4.8 (4.3, 5.5) | 4.2 (3.6, 5.7) | 0.06 |
| Lactate | 980 (759, 1086) | 1016 (821, 1333) | 0.12 |
| Pyruvate | 93.0 (84.5, 110.0) | 107.1 (85.8, 146.0) | 0.08 |
| Malate | 3.1 (2.7, 3.6) | 2.7 (2.3, 3.4) | 0.12 |
| Citrate | 85.1 (72.6, 98.7) | 72.9 (65.3, 89.3) | 0.08 |
| P-values less than 0.05 are shown in bold. | | | |

| **Supplementary Table 2. Unadjusted comparison of all measured plasma amino acids, acylcarnitines, and organic acids between obese HIV-infected subjects and obese HIV-negative controls** | | | |
| --- | --- | --- | --- |
| **Plasma metabolite** | **Obese HIV-infected (n=35)** | **Obese HIV-negative controls (n=30)** | **p-value** |
| **Amino acids, median µM (IQR)** | | | |
| Alanine | 278 (255, 319) | 280 (213, 349) | 0.58 |
| Arginine | 29.4 (22.3, 32.6) | 29.1 (23.5, 36.2) | 0.37 |
| Asparagine | 32.2 (28.4, 34.8) | 31.7 (27.2, 38.3) | 0.81 |
| Aspartic acid | 4.61 (3.34, 5.90) | 3.55 (3.14, 5.02) | 0.18 |
| Citrulline | 29.7 (25.2, 34.1) | 29.3 (23.1, 33.1) | 0.48 |
| Glutamic acid | 43.0 (35.0, 65.7) | 37.5 (28.2, 49.4) | 0.17 |
| Glutamine | 344 (299, 373) | 350 (266, 387) | 0.77 |
| Glycine | 220 (177, 262) | 218 (164, 290) | 0.75 |
| Histidine | 69.7 (62.9, 73.1) | 64.6 (57.2, 74.5) | 0.22 |
| Isoleucine | 55.7 (50.8, 64.0) | 54.6 (45.4, 65.2) | 0.56 |
| Leucine | 105.3 (87.8, 115.6) | 101.5 (93.7, 117.9) | 0.99 |
| Lysine | 202 (182, 218) | 197 (180, 284) | 0.72 |
| Ornithine | 39.9 (34.0, 48.1) | 43.0 (34.4, 48.4) | 0.83 |
| Phenylalanine | 41.7 (38.0, 46.7) | 41.2 (37.5, 46.8) | 0.77 |
| Proline | 137.5 (112.1, 155.5) | 127.7 (98.0, 159.0) | 0.56 |
| Serine | 70.2 (58.7, 83.8) | 75.8 (61.8, 92.8) | 0.22 |
| Threonine | 94.4 (81.4, 117.7) | 120.0 (99.2, 149.5) | **0.02** |
| Tryptophan | 32.2 (26.6, 35.2) | 33.4 (28.0, 39.8) | 0.52 |
| Tyrosine | 49.6 (43.0, 55.0) | 45.2 (30.4, 56.6) | 0.20 |
| Valine | 186 (161, 210) | 178 (153, 212) | 0.50 |
| **Acylcarnitines, median µM (IQR)** | | | |
| C2 | 6.14 (5.11, 8.64) | 6.07 (5.06, 7.33) | 0.51 |
| C3 | 0.32 (0.24, 0.40) | 0.26 (0.20, 0.33) | 0.11 |
| C4 Butyryl | 0.075 (0.067, 0.101) | 0.085 (0.059, 0.108) | 0.70 |
| C4 Isobutyryl | 0.052 (0.037, 0.081) | 0.069 (0.048, 0.085) | 0.21 |
| C4-DC Succinyl | 0.0079 (0.0068, 0.0111) | 0.0079 (0.0054, 0.0097) | 0.55 |
| C4-OH Butyryl | 0.0058 (0.0050, 0.0144) | 0.0060 (0.0050, 0.0128) | 0.67 |
| C5 2-Methylbutyryl | 0.034 (0.025, 0.042) | 0.033 (0.025, 0.038) | 0.67 |
| C5 Isovaleryl | 0.069 (0.050, 0.087) | 0.071 (0.052, 0.086) | 0.95 |
| C5 Valeryl | 0.0030 (0.0025, 0.0044) | 0.0030 (0.0025, 0.0038) | 0.77 |
| C5:1 | 0.0041 (0.0032, 0.0064) | 0.0037 (0.0032, 0.0052) | 0.27 |
| C5-OH | 0.0075 (0.0055, 0.0098) | 0.0062 (0.0056, 0.0076) | 0.13 |
| C6 | 0.032 (0.027, 0.040) | 0.030 (0.025, 0.037) | 0.20 |
| C8 | 0.096 (0.062, 0.124) | 0.079 (0.069, 0.093) | 0.41 |
| C8-OH | 0.015 (0.012, 0.022) | 0.011 (0.010, 0.017) | **<0.01** |
| C10 | 0.081 (0.062, 0.119) | 0.066 (0.053, 0.086) | 0.10 |
| C10-OH | 0.048 (0.036, 0.063) | 0.027 (0.023, 0.042) | **<0.001** |
| C12 | 0.042 (0.031, 0.052) | 0.033 (0.024, 0.040) | **0.02** |
| C14:1 | 0.054 (0.045, 0.079) | 0.048 (0.034, 0.063) | **0.04** |
| C14:2 | 0.026 (0.020, 0.038) | 0.019 (0.013, 0.028) | **<0.01** |
| C14 | 0.018 (0.015, 0.024) | 0.015 (0.013, 0.018) | **0.02** |
| C16:2 | 0.0083 (0.0056, 0.0101) | 0.0056 (0.0049, 0.0079) | **<0.05** |
| C16 | 0.074 (0.062, 0.092) | 0.061 (0.053, 0.075) | **0.01** |
| C18:1 | 0.113 (0.097, 0.133) | 0.097 (0.082, 0.130) | 0.15 |
| C18:2 | 0.087 (0.071, 0.107) | 0.071 (0.061, 0.089) | **<0.05** |
| C18 | 0.032 (0.028, 0.035) | 0.025 (0.022, 0.029) | **<0.001** |
| **Organic acids, median µM (IQR)** | | | |
| 2-hydroxybutyrate | 36.8 (28.5, 47.7) | 32.8 (29.1, 40.1) | 0.20 |
| 3-hydroxybutyrate | 54.4 (29.0, 119.9) | 44.9 (34.2, 87.0) | 0.94 |
| α-ketoglutarate | 4.54 (4.09, 5.34) | 5.40 (4.20, 6.23) | 0.23 |
| Lactate | 988 (835, 1227) | 899 (745, 1113) | 0.14 |
| Pyruvate | 108.3 (93.0, 136.4) | 73.7 (63.2, 96.9) | **<0.001** |
| Malate | 2.78 (2.48, 3.39) | 2.78 (2.50, 3.18) | 0.67 |
| Citrate | 79.4 (70.5, 93.3) | 76.1 (58.6, 89.7) | 0.25 |
| P-values less than 0.05 are shown in bold. | | | |
